# A Human Artificial Chromosome Recapitulates the Metabolism of Native Telomeres in Mammalian Cells

**DOI:** 10.1371/journal.pone.0088530

**Published:** 2014-02-18

**Authors:** Michihito Wakai, Satoshi Abe, Yasuhiro Kazuki, Mitsuo Oshimura, Fuyuki Ishikawa

**Affiliations:** 1 Department of Gene Mechanisms, Graduate School of Biostudies, Kyoto University, Yoshida-Konoe-cho, Sakyo-ku, Kyoto, Japan; 2 Department of Biomedical Science, Institute of Regenerative Medicine and Biofunction, Graduate School of Medical Science, Tottori University, Yonago, Tottori, Japan; 3 Chromosome Engineering Research Center, Tottori University, Tottori, Japan; INSERM UMR S_910, France

## Abstract

Telomeric and subtelomeric regions of human chromosomes largely consist of highly repetitive and redundant DNA sequences, resulting in a paucity of unique DNA sequences specific to individual telomeres. Accordingly, it is difficult to analyze telomere metabolism on a single-telomere basis. To circumvent this problem, we have exploited a human artificial chromosome (HAC#21) derived from human chromosome 21 (hChr21). HAC#21 was generated through truncation of the long arm of native hChr21 by the targeted telomere seeding technique. The newly established telomere of HAC#21 lacks canonical subtelomere structures but possesses unique sequences derived from the target vector backbone and the internal region of hChr21 used for telomere targeting, which enabled us to molecularly characterize the single HAC telomere. We established HeLa and NIH-3T3 sub-lines containing a single copy of HAC#21, where it was robustly maintained. The seeded telomere is associated with telomeric proteins over a length similar to that reported in native telomeres, and is faithfully replicated in mid-S phase in HeLa cells. We found that the seeded telomere on HAC#21 is transcribed from the newly juxtaposed site. The transcript, HAC-telRNA, shares several features with TERRA (telomeric repeat-containing RNA): it is a short-lived RNA polymerase II transcript, rarely contains a poly(A) tail, and associates with chromatin. Interestingly, HAC-telRNA undergoes splicing. These results suggest that transcription into TERRA is locally influenced by the subtelomeric context. Taken together, we have established human and mouse cell lines that will be useful for analyzing the behavior of a uniquely identifiable, functional telomere.

## Introduction

The telomere nucleoprotein complex protects linear chromosomal ends from degradation and fusion in eukaryotes. Telomere DNA is composed of a duplex array of 5′-TTAGGG-3′/5′-CCCTAA-3′ that runs from a few to a hundred kilobases (kb) in mammals. The guanine-rich strand (TTAGGG repeats) and the cytosine-rich strand (CCCTAA repeats) are called the G-strand and C-strand, respectively. A terminal 50 to 200-nt stretch of the G-strand protrudes from the 3′-end of the chromosome as single-stranded DNA (G-tail). Telomere repeats recruit a six-protein complex called shelterin, consisting of TRF1, TRF2, Rap1, TIN2, TPP1, and POT1 [1]. Of these, TRF1 and TRF2 directly bind to the double-stranded (ds) telomere repeat DNA, and POT1 binds to the single-stranded (ss) G-tail. Shelterin plays a pivotal role in maintaining telomere integrity. First, it prevents telomeres from eliciting a DNA damage response and activating DNA repair that would otherwise lead to mitotically catastrophic end-to-end fusions [2], [3]. Second, it has been recently revealed that TPP1 recruits telomerase, a specialized reverse transcriptase that counteracts replication-dependent shortening of telomeres [4], [5]. Paradoxically, telomeres must protect themselves, presumably by barring access to *trans*-factors such as DNA damage checkpoint sensors and DNA repair enzymes, yet telomeres must accommodate *trans*-factors necessary for maintaining telomere integrity, such as the DNA replication apparatus. Therefore, it is supposed that telomeres dynamically change their structure and function during the cell cycle, differentiation, and ageing. From this perspective, it is important to understand when and how telomeres are replicated in S phase.

Telomere DNAs as a whole are replicated throughout S phase [6], [7]. However, individual telomeres are replicated at different and specific time-windows in S phase [8], [9]. We previously measured the replication timing of one seeded telomere in HeLa cells, and found that the particular single telomere is replicated in a 4-hr interval peaking at 4 hr post-release of the G1/S cell cycle block [10].

Telomere DNA is transcribed by RNA polymerase II (Pol II) to produce a non-coding telomeric repeat-containing RNA (TERRA) initiating from subtelomeric regions to G-strands in a wide range of species [11], [12]. Some TERRAs are generated from a single transcription start site 1–2 kb proximal to the telomere repeats [13]. A specific CpG-rich element with promoter activity, along with CTCF and cohesin that occupy upstream sites, stimulates transcription toward the telomere in half of human chromosomes [14], while it is absent in the rest [13].

TERRA often lacks a poly(A) sequence, which is unusual for a Pol II-transcript, and includes a short UUAGGG tract (≈200 nt), leading to rapid turnover [15]. Poly (A)^−^ TERRA associates with telomere chromatin [15]. Notably, depletion of TERRA from telomeres provokes persistent DNA damage that results in aberrant metaphase telomeres, substantiating its protective function as a constitutive factor of telomeres [16], [17]. Consistent with this notion, TERRA excludes RPA from, and promotes POT1 loading onto G-tails in late S phase to ensure the end protective state [18].

It is suggested that changes in TERRA levels are accompanied by epigenetic modifications at telomeres during developmental stages and de-differentiation into iPS cells [12], [19]. However, the molecular readers that appropriately interpret the transcriptional status of TERRA into telomere homeostasis have remained elusive.

One central difficulty in mammalian telomere biology derives from the highly repetitive and polymorphic nature of the DNA sequences at the telomere and subtelomere. This makes it challenging to pursue the dynamics of individual telomeres at high spatio-temporal resolution. We addressed this problem by exploiting telomere seeding, in which transfection of cloned telomere DNA repeats (telomere-targeting vector) occasionally establishes a *de novo* telomere at the distal end of the integration site [20]–[22]. To avoid uncertainty and selection bias inherent to the random integration of the telomere-targeting vector, we instead utilized a gene-targeting technique in DT40 immortalized chicken lymphoblasts to site-specifically induce telomere seeding on a particular human chromosome [23]. Here we used microcell-mediated chromosome transfer to establish a human artificial chromosome (HAC) that is retained in mammalian cells. We analyzed the replication, transcription, and the binding of telomere-specific proteins at the seeded telomere and subtelomere. Investigations focusing on a single telomere in mammalian cells will be invaluable to fully understand the dynamic nature of telomere metabolism.

## Materials and Methods

### Cell lines and cell culture

A DT40 microcell hybrid cell line (DT40(#21)puro339) containing a q-arm truncate of human chromosome 21 (hChr21), which in this paper is called HAC#21, was previously established [23]. As described, a DT40 hybrid carrying a single copy of hChr21, DT40(#21), was used to obtain HAC#21 by integration of the telomere-seeding vector, pBS-TEL/Puro/21q. DT40 hybrid cells were cultured at 40°C in Roswell Park Memorial Institute (RPMI) 1640 medium (Invitrogen) containing 10% fetal bovine serum (FBS) (JRH Biosciences), 1% chicken serum (Invitrogen), 50 µM 2-mercaptoethanol (Sigma) and penicillin-streptomycin (PC-SM) (Gibco), under selection with 0.3 µg/ml puromycin (Sigma). HeLa and NIH-3T3 cells, and their HAC#21-transferred derivatives were maintained in Dulbecco's modified Eagle's medium (DMEM) (Nissui) supplemented with 10% FBS and PC-SM.

### Microcell-mediated chromosome transfer (MMCT)

HAC#21 was transferred from DT40(#21)puro339 to HeLa or NIH-3T3 cells by MMCT, following an established protocol [23]. Briefly, the donor cells were cultured with medium containing 0.05 µg/ml colcemid (Gibco) and 20% FBS for 12 hrs to allow microcell formation. In FBS-depleted medium with 0.01 mg/ml cytochalasin B (Sigma), microcells were harvested by centrifuging 1×10^9^ DT40 hybrid cells attached on flasks (Nalgene Nunc) coated with poly-**l**-lysine (Sigma) for 1 hr at 8,000 rpm, at 34°C. After three rounds of filtration to avoid contamination of the host cells, microcells were gently suspended in DMEM containing 0.05 mg/ml phytohemagglutinin P. Microcells were then dropped onto about 3×10^6^ attached HeLa or NIH-3T3 target cells that had been pretreated immediately beforehand with 47% polyethylene glycol 1000 (WAKO) for exactly 1 min. HAC#21-carrying clones were selected with 0.5–1.5 µg/ml puromycin.

### Fluorescence *in situ* hybridization (FISH)

Metaphase spreads were prepared by following a standard protocol. For FISH on interphase chromosomes, cells were pre-permeabilized on ice for 5 min with cytoskeleton (CSK) buffer (20 mM HEPES-KOH, pH 7.4, 50 mM NaCl, 3 mM MgCl_2_, and 0.3 M sucrose) containing 0.1% Triton X-100, fixed on ice for 10 min in PBS with 3.7% formaldehyde and 0.25% glutaraldehyde, and permeabilized with CSK buffer with 0.5% Triton X-100. Hybridization was performed at 37°C overnight in 50% formamide, 2× SSC (0.3 M NaCl, 30 mM sodium citrate, pH 7.0), 1 mg/ml salmon sperm DNA, 10% dextran sulfate, and 5× Denhardt's (0.1% Ficoll, 0.1% polyvinylpyrolidone, and 0.1% bovine serum albumin). The probes were labeled by nick-translation (Roche) with digoxigenin-conjugated dUTP using α-satellite DNA clone p11-4 [24], which is derived from centromere DNA on human chromosome 21. A biotinylated oligonucleotide with hChr21/13-specific α-satellite sequence, 5′-TGTGTACCCAGCCAAAGGAGTTGA-3′, was used for interphase FISH analysis [25]. The digoxigenin signal was detected with an anti-digoxigenin–rhodamine complex (Roche). The biotin signal was detected with a streptavidin-Alexa488 complex (Molecular Probes) following sequential layers of streptavidin-Alexa488 and biotinylated anti-avidin D antibody (Vector). After DNA-staining with Hoechst 33342 (Molecular Probes), the slides were mounted with VectaShield without dye (Vector Laboratories), and analyzed with a Delta Vision microscope (Applied Precision). Images taken in Z-sections were deconvoluted using SoftWoRx (Applied Precision).

### Southern hybridization

Southern blot was performed as described [26], with slight modification. Briefly, genomic DNA was restricted, separated on a 0.7% agarose gel, depurinated, then transferred to Hybond N^+^ membranes (Amersham) by alkaline transfer. Blots were UV-crosslinked (Stratagene) and hybridized in Church buffer overnight at 55–65°C depending on probes. The probes were labeled by random priming (Roche) with 50 µCi of α^32^P-dCTP. Probe DNA specific for plasmid sequence was obtained by PCR using a cloned plasmid template, or a fragment excised from *Pgk*-puro cloned in pBluescript II SK(+). To prepare a genomic probe, a specific PCR-amplified genomic fragment was cloned, sequence-verified, excised, and gel-purified. After hybridization, the membrane was washed in 1% SDS, 40 mM sodium phosphate (pH 7.2), and 1 mM EDTA, and exposed to a storage phosphor screen (FUJIFILM) which was then visualized with a Typhoon 9400 phosphor image analyzer (GE Healthcare).

### BAL31 assay

Genomic DNA was incubated with 0.033 U/µl BAL31 (TaKaRa), and at designated time points aliquots were removed, and the reaction was stopped by adding 20 mM EGTA. After phenol/chloroform/isoamyl alcohol extraction and alcohol precipitation, DNA was analyzed by Southern hybridization.

### Cell-cycle synchronization and replication timing analysis

HAC#21-HeLa cells were treated with 2 mM thymidine for 16 hrs, released into normal medium for 11 hrs, and then treated with 1 µg/ml aphidicolin for 14 hrs. Following release, cells were pulse-labeled with 50 µM 5-bromo-2′-deoxyuridine (BrdU) for one-hour intervals, chased with normal medium, and harvested 9 hrs post release. For monitoring cell cycle progression, a parallel sample of cells was harvested at each time point, stained with propidium iodide, and analyzed for DNA content by FACS Calibur (BD). For replication analysis, genomic DNA, extracted using a Wizard Genomic DNA Extraction kit (Promega), was sonicated to a few hundred bp using a Bioruptor (BioRad). 2 µg of the sonicated DNA and 2 ng of BrdU-substituted/sonicated *Escherichia coli* DNA were heat-denatured in a mixture and immunoprecipitated with 5 µg of anti-BrdU antibody (Roche, BMC9318) in 100 µl of IP buffer (0.05% Triton X-100, 140 mM NaCl, and 10 mM sodium phosphate, pH 7.2) for 30 min at room temperature (RT), and further rotated for 30 min with 20 µl of Dynal M-280 sheep anti-mouse IgG (Invitrogen). The beads were washed four times each for 5 min with 500 µl of IP buffer, eluted twice in 100 µl of 1% SDS/TE at 100°C for 3 min, and DNA was de-proteinized, purified, and subjected to real-time PCR as in ChIP. The graphs and regression curves were drawn with KaleidaGraph (Hulinks).

### Chromatin immunoprecipitation (ChIP)

ChIP was done essentially as described [26], with slight modifications. Briefly, 1×10^7^ cells suspended in phosphate-buffered saline (PBS) (Nissui) were fixed by adding 1% formaldehyde for 10 min at RT. After the reaction was quenched with 125 mM glycine, the fixed cells were washed in PBS and lysed on ice for 10 min in 100 µl of Cell Lysis buffer (1% SDS, 10 mM EDTA, 50 mM Tris, pH 8.0, and protease inhibitors (Roche)). The lysates were diluted with 4-volume Cell Lysis buffer without SDS, and sonicated with a Bioruptor (BioRad) at 4°C, 10 or 15 min in total, with equal on/off switching of 15 sec. Cleared sonicates were diluted 10-fold with 1% Triton X-100, 150 mM NaCl, 2.5 mM KCl, 5 mM Na_2_HPO_4_, 1.5 mM KH_2_PO_4_, 10 mM EDTA, 25 mM Tris, pH 7.6, 10% glycerol, 1 mM dithiothreitol, and protease inhibitors (Roche), then mixed with each antibody and 40 µl of Dynal M-280 sheep anti-rabbit IgG or sheep anti-mouse IgG, or 80 µl of Dynal M-450 sheep anti-rat IgG magnetic beads (Invitrogen), and rotated at 4°C overnight. Beads were washed successively with 750 µl of low salt NET buffer (0.5% NP-40, 1 mM EDTA, 1 mM EGTA, 150 mM NaCl, 5 mM MgCl_2_, and 50 mM Tris, pH 8.0), high salt NET buffer (NET buffer with 300 mM NaCl), LiCl buffer (10 mM Tris, pH 8.0, 250 mM LiCl, 1 mM EGTA, 0.5% NP-40 and 0.5% Triton X-100), and TE (10 mM Tris, pH 8.0, 1 mM EDTA). Each wash was for 5 min at 4°C. The number of repetitions of each wash step varied, depending on the antibodies used. The DNA-protein complex was eluted from the beads in 1% SDS, 0.1% NaHCO_3_ at 68°C for 10 min, and de-crosslinked at 65°C overnight after addition of an equal volume of 300 mM NaCl, 100 µg/ml RNase A. Co-precipitated DNA was extracted with phenol/chloroform/isoamyl alcohol, ethanol-precipitated with 2 µl of 20 mg/ml glycogen (Nacalai) at −80°C, and dissolved in TE. 5% of the DNA was used for real-time PCR, performed with an ABI PRISM 7000 or StepOne Plus (Applied Biosystems), using Power SYBR green PCR master mix (Applied Biosystems). The PCR program consisted of 45 cycles of 95°C, 5 sec, and 60°C, 31 sec.

### Antibodies

For ChIP, normal rabbit IgG (Santa Cruz, sc-2027), anti-histone H3 (Abcam, ab1791), anti-TRF1 (in-house), anti-TRF2 (Millipore, #05-521), anti-TPP1 (in-house) and anti-Pol II C-terminus domain (CTD) (Abcam, ab5408; clone 4H8) were used. Anti-phospho Ser-2 CTD (clone 3E10) and anti-phospho Ser-5 CTD were previously described [27]. Immunoblotting utilized anti-nucleolin (Abcam, ab13541), anti-tubulin (Sigma, T3526), anti-hnRNPA1 (Abcam, ab10685) anti-histone H2B (Millipore, #07-371), and anti-GAPDH (Millipore, clone 6C5).

### RNA preparation and RT-PCR/RT-real-time PCR

To extract RNA in the nuclear fraction, cells were trypsinized, washed with PBS, and extracted with 250 µl of NP-40 buffer (10 mM Tris, pH 7.5, 10 mM NaCl, 3 mM MgCl_2_, 0.5% NP-40, 40 U/µl RNasin (Promega), and 1 mM dithiothreitol) on ice for 10 min. Nuclei were pelleted by centrifugation at 500× *g* for 5 min at 4°C, washed once with NP-40 buffer, and processed as in total RNA extraction. Total RNA was extracted using an RNeasy Mini kit (Qiagen) with on-column DNase I digestion following the manufacturer's protocol. 1 or 2 µg of RNA was reverse-transcribed with random nonamers or a telomere-specific 5′-(CCCTAA)_5_-3′ primer at 55°C using AMV reverse-transcriptase XL (TaKaRa). cDNA was subjected to either real-time PCR as described for ChIP analyses, or PCR using LA-*taq* DNA polymerase (TaKaRa).

### Purification of telomeric repeat-containing RNA and Northern hybridization

Total RNA (50 µg) and 5 pmol of biotinylated oligonucleotide containing 5′-(CCCTAA)_5_-3′ repeat were combined with Binding Buffer (10 mM Tris (pH 7.6), 0.5 M NaCl and 1 mM EDTA) in a total volume of 300 µl. The mixture was heated at 65°C for 2 min, then cooled on ice, and added to 10 µg of Dynal M280 Streptavidin magnetic beads. After gentle rotation at RT for 1 hour, the beads were washed three times for 5 min at RT with 300 µl Wash buffer (10 mM Tris-Cl (pH 7.6), 150 mM LiCl, 1 mM EDTA). An aliquot of 1/25 of the washed beads was removed for RT-PCR to check the enrichment of TERRA, and the rest was used for Northern hybridization. For Northern hybridization, the beads complex was heat-denatured and separated on a denaturing gel (1% Seakem GTG agarose, 6% formaldehyde and 1× Goldberg buffer (40 mM MOPS (pH 7.2), 5 mM sodium-citrate, 0.5 mM EDTA)) in 1× Goldberg buffer. The gel was neutralized in 0.25 M ammonium acetate (pH 5.0) and stained with SYBR Gold, then transferred onto Hybond N^+^ membrane in 25 mM sodium phosphate buffer (pH 6.5). The blot was UV-crosslinked at 0.12 J/m^2^ and hybridized with a ^32^P-labeled probe in 7% SDS, 0.5 M sodium phosphate (pH 6.5), 1 mM EDTA overnight at 65°C. The hybridized membrane was washed in 1% SDS, 40 mM sodium phosphate (pH 6.5) and exposed to a phosphor screen. The autoradiograph was analyzed with a Typhoon 9400 phosphor imager and ImageQuant software (GE Healthcare). The densitometric data was graphed using Excel (Microsoft).

### 5′ Rapid amplification of cDNA ends (5′ RACE)

The transcription start site for HAC-telRNA was determined using a SMART RACE cDNA amplification kit (Clontech) according to the manufacturer's protocol. Reverse-transcription was performed with a pBluescript-specific primer using 0.5 µg of RNA from the nuclear fraction, followed by PCR with 45 cycles of 95°C for 15 sec, 60°C for 20 sec, and 72°C for 3 min.

### Subcellular fractionation and quantification of RNA

Cell fractionation was done essentially as described [15]. For quantification of RNA, RNA was purified from a uniform portion of each fraction with an RNeasy Mini kit (Qiagen) as described above. cDNAs were then synthesized with a mixture of gene-specific and 5′-(CCCTAA)_5_-3′ primers, and subjected to real-time PCR analyses as described in ChIP.

### siRNA

A cocktail of siRNA oligonucleotides against human *TRF1* (Cosmo Bio, SHF27A-2210-C) or a negative control was transiently transfected into cells using Lipofectamine 2000 (Invitrogen), according to the manufacturers' protocols. Total RNA was prepared from cells 48 hrs post-transfection. RT-PCR gels were stained with SYBR Gold (Invitrogen), and signals were quantified by Typhoon 9400 and ImageQuant software.

### Primer sequences

PCR primers used for probe construction in Southern hybridization are as follows:

pBS-fwd: 5′-TCAAGCTTATCGATACCGTCGACC-3′

pBS-rvs: 5′-GAGTACTCAACCAAGTCATTCTGAG-3′

21p-fwd: 5′-CTGGCACTCCTGCATAAACA-3′

21p-rvs: 5′-TCTGTGTTCCCCTTCTCTGA-3′

21q-fwd: 5′-AGAGCTCTCTTGCTTGAAGATTGG-3′

21q-rvs: 5′-CTTCATGGCACAGACTCTGCACAG-3′

PCR primers used for real-time PCR or oligonucleotide probes in ChIP experiments:

x-fwd: 5′-TCAAGCTTATCGATACCGTCGACC-3′

x-rvs: 5′-GTGCTGCAAGGCGATTAAGT-3′

y-fwd: 5′-TAAACAAATAGGGGTTCCG-3′

y-rvs: 5′-TAACGCTTACAATTTAGGTG-3′

z-fwd: 5′-GGCTTGTACTCGGTCATGGT-3′

z-rvs: 5′-CTTTCGACCTGCATCCATCT-3′

w-fwd: 5′-AGGCCAGGAACCGTAAAAAG-3′

w-rvs: 5′-GATTTTTGTGATGCTCGTCA-3′

ACTG1-fwd: 5′-CTCTCGCACTCTGTTCTTCC-3′

ACTG1-rvs: 5′-GCTCACCGGCAGAGAAA-3′

ADH5-fwd: 5′-GCATAATTGAGCCTACGCC-3′

ADH5-rvs: 5′-GCAGAGGTGTTTGTTACGTG-3′

γ-globin-fwd: 5′-TCTACCCATGGACCCAGAGGT-3′

γ-globin-rvs: 5′-CCACATGCAGCTTGTCACAGT-3′

(CCCTAA)_5_: 5′-CCCTAACCCTAACCCTAACCCTAACCfCTAA-3′

Chr21/Chr13 alphoid: 5′-TGTGTACCCAGCCAAAGGAGTTGA-3′

Oligonucleotide DNA used for pull-down of telomeric repeat-containing RNA:

5′-biotin-TGCTCCGTGCATCTGGCATCCCCTAACCCTAACCCTAACCCTAACCCTAA-3′

Gene-specific RT primers in RT-PCR/RT-real-time PCR experiments are as follows:

Telomeric repeat-containing RNA:

5′-TGCTCCGTGCATCTGGCATCCCCTAACCCTAACCCTAACCCTAACCCTAA-3′ or 5′-CCCTAACCCTAACCCTAACCCTAACCCTAA-3′

GAPDH: 5′-TTACTCCTTGGAGGCCATGTGG-3′

U1: 5′-CATCCGGAGTGCAATGGATA-3′

NEAT1: 5′-GTTTAGAACTCAAACTTTATTTGTGC-3′

XIST: 5′-GGAACAATGAAGAGCTTGAC-3′

PCR primers in RT-PCR/RT-real-time PCR experiments are as follows:

5′ RACE for HAC-telRNA: 5′-GCAGTGCTGCCATAACCATGAGTG-3′

HAC-telRNA (unspliced and spliced) fwd: 5′-TCAAGCTTATCGATACCGTCGACC-3′

HAC-telRNA (unspliced and spliced) rvs: 5′-GTGCTGCAAGGCGATTAAGT-3′

HAC-telRNA (variant 1) fwd: 5′-CTGCGCCTTATCCGTTGC-3′

HAC-telRNA (variant 1) rvs: 5′-GATAAATCTGGAGCCGGTGA-3′

HAC-telRNA (variant 1 and 2) fwd: 5′-GCGCTTTCTCATAGCTCACG-3′

HAC-telRNA (variant 1 and 2) rvs: 5′-ACGACGGGGAGTCAGGCAACGG-3′

Xp-Yp TERRA fwd: 5′-CCCTCTGAAAGTGGACCTAT-3′

Xp-Yp TERRA rvs: 5′-GAGTGAAAGAACGAAGCTTCC-3′

15q-TERRA fwd: 5′-CAGCGAGATTCTCCCAAGCTAAG-3′

15q-TERRA rvs: 5′-AACCCTAACCACATGAGCAACG-3′

18S-fwd: 5′-CTTAGAGGGACAAGTGGCG-3′

18S-rvs: 5′-ACGCTGAGCCAGTCAGTGTA-3′

GAPDH-fwd: 5′-TGCACCACCAACTGCTTAGC-3′

GAPDH-rvs: 5′-GGCATGGACTGTGGTCATGA-3′

c-Myc-fwd: 5′-CTCCTACGTTGCGGTCACAC-3′

c-Myc-rvs: 5′-CCGGGTCGCAGATGAAACTC-3′

NEAT1-fwd: 5′-GACTTGGGGATGATGCAAAC-3′

NEAT1-rvs: 5′-TCACAACAGCATACCCGAGA-3′

TRF1-fwd: 5′-TCCTCTGCCTCTCTCTTTGC-3′

TRF1-rvs: 5′-GGTTTTTCCTGCTGCAATTC-3′

U1-fwd: 5′-TACCTGGCAGGGGAGATACC-3′

U1-rvs: 5′-CATCCGGAGTGCAATGGATA-3′

WASH-fwd: 5′-AGCCAAGGTGGGGACTTGAT-3′

WASH-rvs: 5′-ACCAGCCCCAGGTCCTTTC-3′

XIST-fwd: 5′-AGCTTGACGTGTGGTGGTTG-3′

XIST-rvs: 5′-AGGTGGAGATGGGGCATGAG-3′

## Results

### HeLa and NIH-3T3 subclones that contain an artificial chromosome

**Figure 1:**
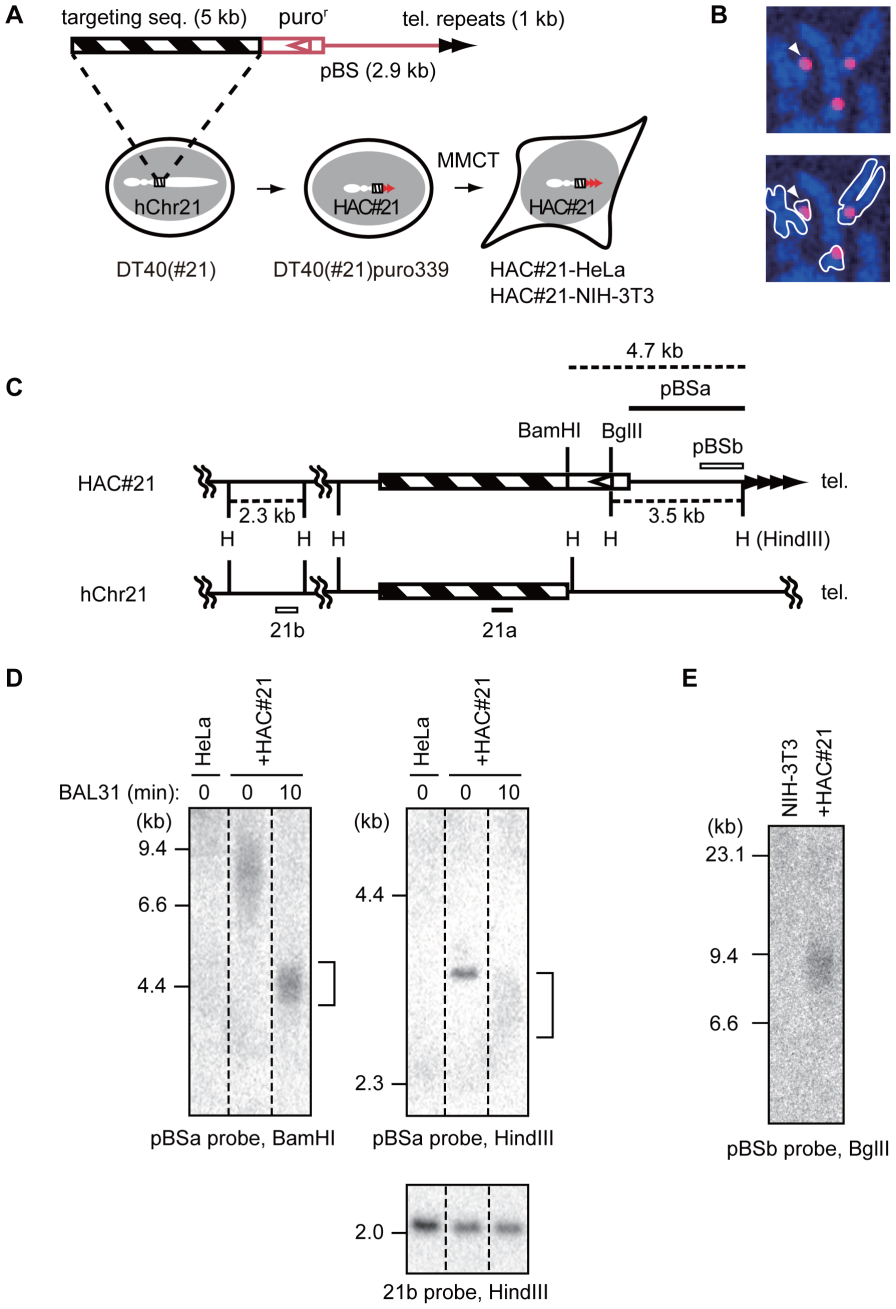
Experimental design of the study. A. The telomere-targeting vector containing a hChr21q-homologous region (striped) was targeted to the long arm of hChr21 in DT40 hybrid cells (left panel). Puro-resistant clones that possessed a mini-chromosome HAC#21 with a *de novo* telomere were obtained (red triangles, middle panel). HAC#21 was transferred to HeLa or NIH-3T3 cells (right) to produce HAC#21-HeLa and HAC#21-NIH-3T3 cells, respectively, by microcell-mediated chromosome transfer. HAC#21 contains vector backbone-derived unique DNA sequences in host cells, as highlighted in red in the plasmid map. B. Metaphase spreads of HAC#21-HeLa chromosomes were hybridized with an alphoid DNA specific to hChr21 and hChr13 (red), and stained for DNA (blue). In addition to the host chromosomes, a small-sized putative mini-chromosome hybridized with the probe, and was consistently spatially distinct from host chromosomes (arrowhead). C. Restriction map of hChr21 versus the presumptive HAC#21 indicating the positions of probes (bars). D. BAL31 sensitivity. Genomic DNAs obtained from indicated cells were treated for 0 min (0) or 10 min (10) with exonuclease BAL31, and subjected to Southern hybridization using indicated probes. +HAC#21 indicates HAC#21-HeLa DNA. The vector fragments detected with the targeting vector-specific probe pBSa (a smear in top left and a band in top right) were sensitive to BAL31 treatment (bracket), whereas a fragment internal to hChr21 was resistant (bottom, 21b). The stronger intensity of the 21b-positive band in lane 1 compared to lanes 2 and 3 was due to overloading of HeLa DNA compared to HAC#21-HeLa DNA. E. Telomere length of HAC#21 in NIH-3T3 cells. The vector fragment containing telomere repeats was detected in BglII digests of DNAs obtained from HAC#21-NIH-3T3 cells (+HAC#21) by Southern hybridization using a pBSb probe.

We have previously reported a human artificial chromosome (HAC) maintained in chicken DT40 cells [23]. Briefly, a human chromosome 21 (hChr21) that possessed a drug selection marker was transferred by microcell-mediated chromosome transfer (MMCT) from mouse A9 hybrid host cells containing hChr21 to DT40 cells, a cell line highly proficient for DNA homologous recombination. A resulting stable cell line, DT40#21, contained a single copy of hChr21. We transfected a telomere-targeting vector to DT40#21 cells (Fig. 1A). The linearized pBluescript-based vector contained 1-kb telomere DNA repeats at one end for induction of *de novo* telomere formation, and a puromycin resistance gene for selection of transfected clones (Fig. 1A). At the other end of the vector was a 5-kb sequence of the native long arm of hChr21 (hChr21q) that targeted integration of the vector at this particular site (nt 15,114,270–15,119,260 on RefSeq (GRCh37.p10), located 0.7-Mb distal to the centromere of hChr21). The predicted size of the truncated chromosome was approximately 15 Mb, corresponding to one-third of that of hChr21 (48 Mb, http://www.ncbi.nlm.nih.gov/mapview/maps.cgi?taxid=9606&chr=21). After transfection, we chose a puromycin-resistant DT40 clone (DT40(#21)puro339) carrying a single copy of hChr21 with a truncated hChr21q that had undergone homologous recombination (Fig. 1A), which we hereafter call HAC#21 (human artificial chromosome derived from hChr21).

We transferred HAC#21 from DT40(#21)puro339 cells to HeLa and NIH-3T3 cells via MMCT. In one experiment, microcells harvested from 1×10^9^ DT40 hybrid cells were fused with 3×10^6^ attached HeLa or NIH-3T3 target cells (Fig. 1A). In total, 3 and 38 puromycin-resistant clones were obtained from 1.5×10^7^ cells of HeLa and NIH-3T3 recipient cells (transformation efficiencies, 2×10^−7^ and 2.5×10^−6^/recipient cell) respectively, in five independent experiments. Among these puromycin-resistant clones, we found that 13 out of 22 NIH-3T3 clones showed smeared telomere signals in an experiment similar to Fig. 1E, suggesting that approximately half of the NIH-3T3 clones possessed HAC (a rough estimate of MMCT efficiency was approximately 38×(13/22)/(1×10^9^×5) = 4.5×10^−9^ HAC-positive NIH-3T3 clone/donor DT40(#21)puro339 cell). We further investigated one each from the HeLa and NIH-3T3 clones, as described below (HAC#21-HeLa and HAC#21-NIH-3T3 cells, respectively). We detected HAC#21 in HAC#21-HeLa cells by fluorescence *in situ* hybridization (FISH) using an alphoid DNA probe that detects the hChr21 and hChr13 (human chromosome 13) centromeres (hChr13/hChr21 alphoid probe) [24]. In metaphase spreads of HAC#21-HeLa cells, we consistently detected FISH signals on a small chromosome that appeared as a dot after DNA staining (Fig. 1B, arrowhead), as well as on native hChr21 and hChr13 chromosomes. We examined the copy number of HAC#21 in HAC#21-HeLa cells by hybridizing the hChr13/hChr21 alphoid probe in interphase HeLa cells, and HAC#21-HeLa cells cultured for 6 weeks with or without puromycin selection (Fig. S1A). The probe produced a median number of four independent signals per nucleus in control HeLa cells, whereas five signals were detected in HAC#21-HeLa cells cultured in the presence or absence of puromycin. The single extra FISH signal in cells harboring HAC#21 and cultured without puromycin selection suggests the presence of a single copy of HAC#21 that was stably maintained. Similarly, we detected a single hChr13/hCh21 alphoid sequence-specific signal in individual interphase HAC#21-NIH-3T3 cells, but not in the parental NIH-3T3 cells (data not shown). Taken together, we conclude that a single copy of HAC#21 is maintained in HAC#21-HeLa cells and HAC#21-NIH-3T3 cells, independently of the host chromosomes.

We characterized the structure of the newly formed telomere of HAC#21 by Southern hybridization. A probe corresponding to the region derived from the hChr21-homologous sequence of the targeting vector (21a in Fig. S1B) detected a 6.3-kb band in HindIII digests obtained from both HeLa and HAC#21-HeLa cells, as expected with the native hChr21 (Fig. S1B). In addition, we detected a 7.5-kb band specifically in HAC#21-HeLa cells (Fig. S1B, arrow). The size of the fragment exactly matched the predicted length of a HindIII junction fragment spanning the boundary between hChr21q and the targeting vector-derived sequence (Fig. S1B). The signal of the native hChr21-derived 6.3-kb band was more intense than that of the HAC#21-derived 7.5-kb band. This was probably caused by segmental duplications involving the 6.3-kb fragment in the human genome (found with Chr21q11.1, Chr21q22.1, Chr2, Chr18 and Chr3) [28]. The 7.5-kb band, but not the 6.3-kb band, was also detected with a probe corresponding to the HAC#21-specific puromycin-resistance gene region (puro) (Fig. S1B). In summary, the 7.5-kb band was positive for both hChr21 sequence (probe 21a) and targeting vector-specific sequence (puro), suggesting that the targeting vector had integrated in the predicted region of hChr21q, as expected from the homology-mediated integration.

HeLa and NIH-3T3 cells are telomerase-positive and displayed telomere lengths of several kb up to thirty kb, by terminal restriction fragment analysis (Fig. S1C, lanes P and HeLa). The telomere-containing fragment typically appears as a smeared hybridization signal that is sensitive to BAL31 exonuclease treatment prior to restriction digestion [29]. When genomic DNAs from HAC#21-HeLa cells were treated with BAL31 for 10 min, digested with BamHI, and hybridized with a probe located distal to the most terminal BamHI site on the targeting vector (pBSa in Fig. 1C), accordingly, the smeared HAC#21-specific band migrated faster (Fig. 1D, top left). This result is consistent with the predicted structure of the seeded telomere, in which the probe detects a BamHI fragment containing the telomere repeats. Using HindIII digests, the pBSa probe detected a discrete 3.5-kb band, rather than a smear, as expected from the presence of a HindIII site distal to the region hybridized with the probe (Fig. 1D, top right, 0 min). The 3.5-kb signal became a faster-migrating smear in BAL31-treated DNA compared to untreated DNA, whereas a chromosome-internal fragment on the short arm of hChr21 detected by the 21b probe was BAL31-resistent (Fig. 1D, bottom), again indicating that the HindIII fragment detected by the pBSa probe was localized close to the terminus of HAC#21.

We deduced the length of the seeded telomere of HAC#21 by subtracting the nucleotide length of subtelomeric DNA from that of the terminal restriction fragments. By densitometric analysis, we estimated that the smeared signal in Fig. 1D (BAL31-untreated HAC#21-HeLa, lane 2) ranged from 5.2 to 10.2 kb. The BamHI fragment contained a 4.7-kb segment of subtelomeric DNA (Fig. 1C), leading to an estimated 0.5 to 5.5 kb length of the seeded telomere repeat DNA in HAC#21-HeLa cells. Similarly, in HAC#21-NIH-3T3 cells, by subtracting a 3.5-kb subtelomeric DNA portion from the BglII-digested terminal fragment of HAC#21 (detected by the pBSb probe in Fig. 1E, lane 2), we estimated that the length of the seeded telomere DNAs of HAC#21 ranged from 3.5 to 8.1 kb. These calculations suggest that HAC#21 telomere DNA is longer in HAC#21-NIH-3T3 than in HAC#21-HeLa cells. We produced multiple independent HAC#21-containing NIH-3T3 clones in addition to the clone described above. All examined NIH-3T3 clones showed longer HAC#21 telomere DNA compared to that of HAC#21-HeLa cells, suggesting that the observations of telomere length were not clone-specific among different NIH-3T3 clones. Native telomeres in NIH-3T3 cells are generally longer than in HeLa cells (Fig. S1C). These results suggest that the length of the seeded telomeres was controlled similarly to the native telomeres.

### Telomere regions of HAC#21 are replicated during mid-S phase in HeLa cells

**Figure 2:**
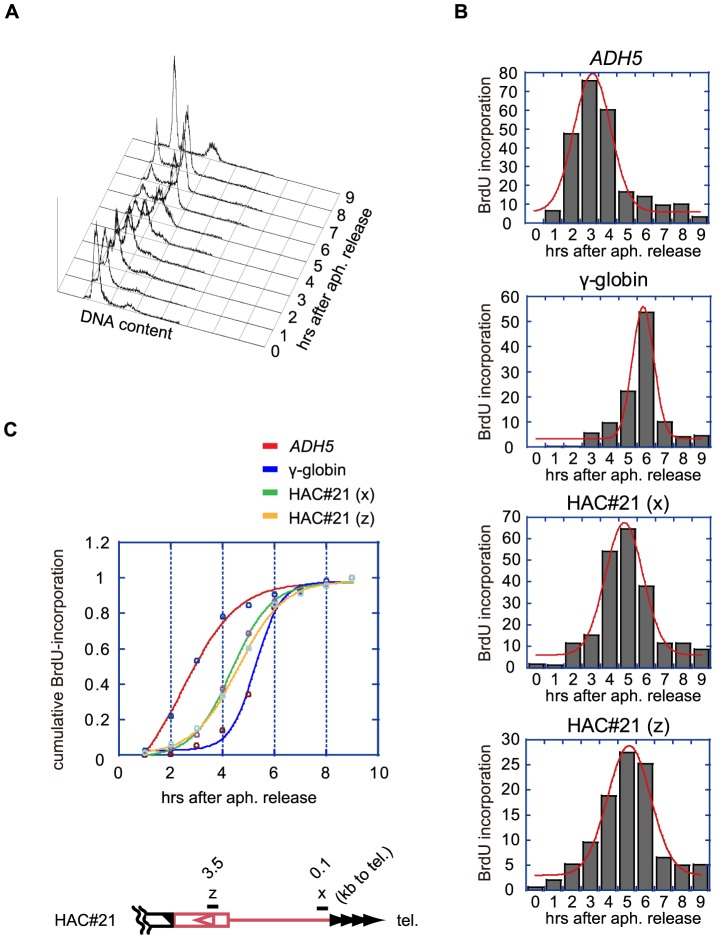
The seeded subtelomere is replicated in mid-S phase in HeLa cells. A. S-phase progression analyzed by flow-cytometry of G1/S-released cells after thymidine-aphidicolin double-block and propidium iodide staining. B. Histograms indicating the amounts of incorporated BrdU at a test locus during each one-hour pulse in S phase, as determined by real-time PCR quantification of immunoprecipitated genomic DNA by anti-BrdU (shown in arbitrary units). x and z indicate PCR regions in the HAC#21 subtelomere shown in C. Each graph is fitted to a normal distribution (red). C. Replication kinetics represented in the cumulative plots of BrdU-incorporation in B, with fitting curves. The distal subtelomeric DNA in HAC#21 (PCR regions x and z) is replicated with timing between early *(ADH5*) and late (γ-globin) in S phase.

To analyze the replication timing of the telomere region in HAC#21, we synchronized HAC#21-HeLa cells at the start of S phase by sequential treatments of cells with thymidine and aphidicolin (Fig. 2A). The synchronous culture was released from the G1-S block by culturing the cells in media without the drug, and split into aliquots. Successive aliquots were labeled with BrdU for 1 hr at consecutive one-hour intervals and chased in the absence of BrdU until 9 hr post-release when most cells exited from S phase. BrdU-labeled nascent DNA was purified by immunoprecipitation with an anti-BrdU antibody, and enrichment of test DNAs was quantified by real-time PCR. All loci analyzed showed a single 1-hr peak interval, during which the BrdU incorporation was greatest (Fig. 2B). It is known that *ADH5* and the gamma-globin gene replicate in early and late S phase, respectively [30], [31]. When cumulative BrdU incorporation was correlated with specific test loci in fractions covering the S phase progression, we found that *ADH5* and gamma-globin loci replicated at early and late S phase, respectively, as expected (Fig. 2C). To examine the replication kinetics of HAC#21, we chose two primer sets, x and z, which were unique to the HAC#21 subtelomere (Fig. 2C). Regions amplified by primer sets x and z are located 0.1-kb and 3.5-kb proximal to the telomere repeat DNAs of the seeded telomere of HAC#21 (regions x and z, respectively). We found that regions x and z synchronously replicated at mid-S phase (Fig. 2B and C). These results indicate that the telomere-proximal region of the seeded telomere of HAC#21, which is devoid of any endogenous hChr21 subtelomere DNA sequence, synchronously replicates at mid-S phase in HAC#21-HeLa cells.

### Spreading of telomere proteins at the telomere-proximal DNA of HAC#21 in HeLa cells

**Figure 3:**
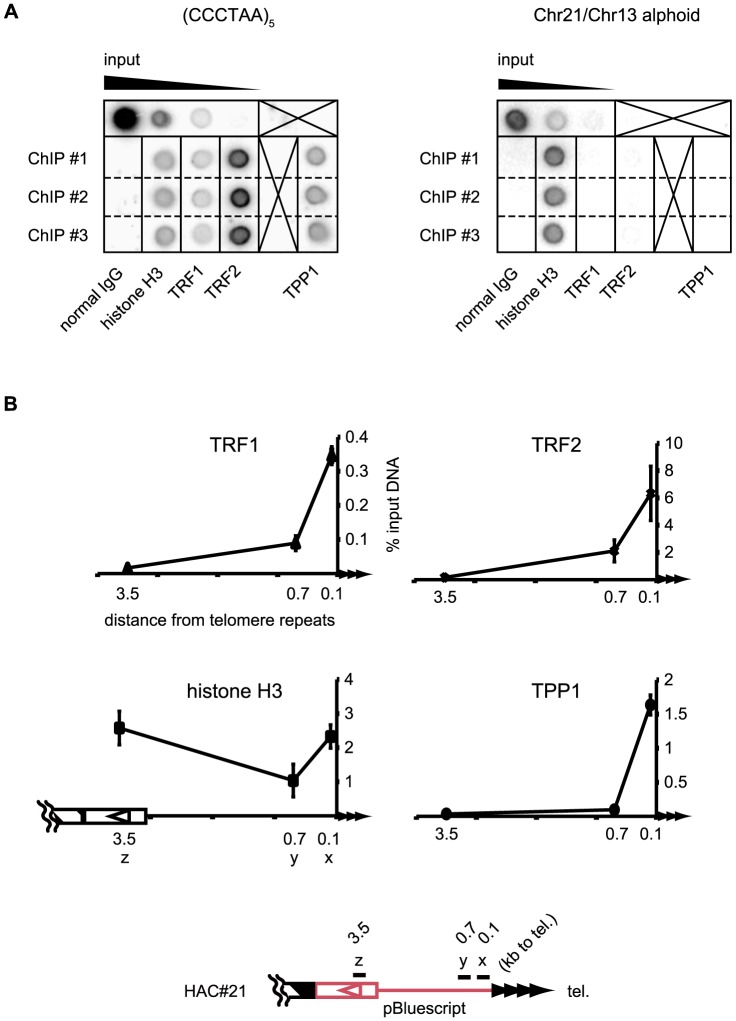
Telomere-binding proteins spread to the seeded subtelomere in HeLa cells. A. Dot blots of chromatin immunoprecipitates indicating specificity of telomere-binding proteins. Left, the telomere-repeat probe. Right, the alphoid probe. Results shown are from three independent immunoprecipitation experiments (ChIP #1-#3). B. The seeded subtelomere was analyzed by real-time PCR after ChIP. The percentage of input DNA precipitated is shown along the distance from telomere repeats in HAC#21 (0.1-, 0.7- and 3.5-kb apart regions were analyzed). Bars indicate s.d. of three independent ChIP experiments.

We conducted chromatin immunoprecipitation (ChIP) to examine if the HAC#21 telomere is associated with shelterin proteins (Fig. 3). First, the specificities of anti-TRF1, TRF2, and TPP1 antibodies were tested by immunoblotting of HeLa cell extracts, and proteins with the expected mobilities of TRF1, TRF2, and TPP1, respectively, were detected (Fig. S2A, arrowheads). DNAs obtained from immunoprecipitates with anti-TRF1, TRF2, and TPP1 antibodies hybridized with a telomere DNA probe, but not with a centromeric alphoid DNA probe. In contrast, both probes detected anti-histone H3 immunoprecipitates, as expected.

We analyzed co-precipitated DNA by real-time PCR, using primer sets x, y, and z (Fig. 3B). The primer set y amplifies a HAC#21 subtelomeric region 0.7-kb proximal to the HAC#21 telomere repeats (region y). In the quantitative ChIP experiment, TRF1, TRF2, and TPP1 were enriched at region x (0.1-kb proximal to the telomere repeats). Interestingly, the enrichments of both TRF1 and TRF2 were significant at region y, albeit at reduced levels compared to region x, while TPP1 appeared absent at region y. In these ChIP experiments, the length of sonicated DNA was largely in the range of less than five hundred bp (Fig. S2B). Given that region y is 0.7 kb centromeric to the telomere repeat DNA array, the positive ChIP data for TRF1 and TRF2 using region y probe suggests that TRF1 and TRF2 bound with the subtelomeric DNA. It is nevertheless possible that the differential enrichment of TRF1 and TRF2 versus TPP1 at region y was caused by different efficiencies of the three antibodies in immunoprecipitation. Alternatively, TRF1 and TRF2 indeed may associate with subtelomeric chromatin more proximally compared to TPP1. It was reported that TRF1 and TRF2 are ten times as abundant as TPP1 in several cancer cell lines [32], and as such it is possible that the different distributions were caused by a mass effect. Importantly, we could not detect any binding of the three telomeric proteins at region z (3.5-kb proximal). Thus, we concluded that TRF1, TRF2, and TPP1 bind to the subtelomeric region of HAC#21. In summary, it was suggested that the shelterin complex is localized at the subtelomere of the HAC#21 telomere. Anti-histone H3 antibody significantly precipitated regions x, y, and z. Therefore, nucleosomes seem to be formed in the vicinity of the telomere repeats.

### Telomeric repeat-containing RNA is transcribed from the seeded subtelomere

**Figure 4:**
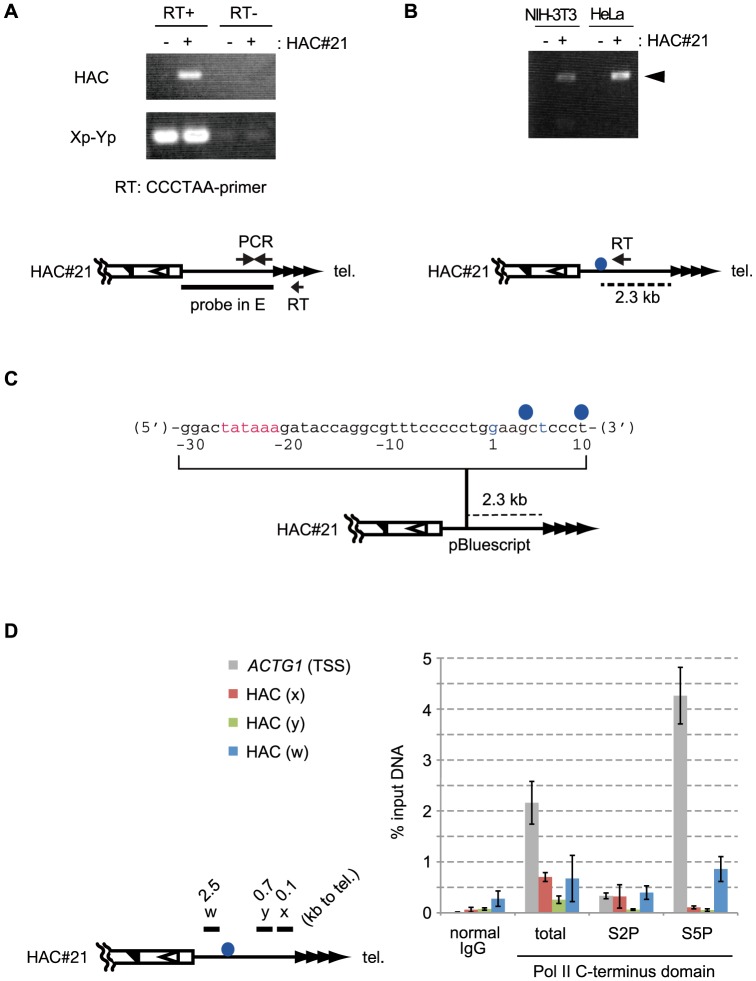
The seeded telomere is transcribed. A. RT-PCR analysis of telomere-repeat-containing transcripts. Nuclear RNAs were converted to cDNA using (CCCTAA)_5_ oligonucleotide DNA with a 5′-end tagging sequence (RT in the lower panel). Unique subtelomeric DNA of HAC#21 (HAC) or chromosome Xp-Yp (Xp-Yp) was amplified (PCR in the lower panel) in a reverse-transcriptase-dependent manner (RT+). RNA was obtained from HeLa (HAC#21−) or HAC#21-HeLa (HAC#21+) cells. B. 5′-RACE of telomere transcripts in HAC#21-HeLa and HAC#21-NIH-3T3 cells. cDNA was produced using a reverse-transcription primer specific to the targeting vector. The PCR product is indicated by an arrowhead in the upper panel. The positions of the primer and deduced transcription start site are indicated in the lower panel by an arrow and a blue circle, respectively. C. Sequence analysis of PCR products in B (arrowhead). Two TSS's (positions labeled by blue circles) were identified for HAC-telRNA. A TATA-like sequence was found immediately upstream of the TSS's (highlighted by red letters). D. Pol II occupancies of the seeded subtelomere determined by ChIP. Antibodies specific to either phosphorylated serine-2 or serine-5 on the C-terminus heptad repeats of the largest subunit of Pol II (S2P and S5P, respectively), and to total Pol II (total) were used. Enrichment of each type of Pol II at the seeded subtelomere (x, y, and w; w is a region 0.2 kb upstream of the TSS's) as well as at the TSS of the γ-actin gene (*ACTG1*) was measured. Bars indicate s.d. of three independent ChIP experiments. The relative positions of PCR regions and TSS's (blue circle) are shown in the left panel.

We investigated whether the seeded telomere of HAC#21 produced any RNA transcripts using RT-PCR experiments. We enriched the nuclear RNA fraction, in which TERRA is exclusively contained [11], [15], and synthesized cDNA using a telomere-specific primer (CCCTAA-primer). This primer is comprised of a 20-nt 5′ unique sequence, used for tagging the cDNA, followed by a 5′-(CCCTAA)_5_-3′ sequence at its 3′ end, so that only RNAs containing telomere repeats will be reverse-transcribed. We prepared specific PCR primers to amplify the telomere-adjacent region unique to either HAC#21 or chromosome Xp-Yp, enabling us to exclusively detect transcripts from respective chromosomal ends. TERRA derived from chromosome Xp-Yp served as a control [11]; the Xp-Yp TERRA signals were detected in an RT-dependent manner in both HAC#21-positive and -negative HeLa cells (Xp-Yp in Fig. 4A). We detected a PCR signal using HAC#21-specific primers only in HAC#21-HeLa, but not in HeLa cells. The signal was dependent on the presence of reverse-transcriptase (RT) in the reaction, demonstrating that the telomere DNA repeats of the seeded HAC#21 telomere were transcribed in HAC#21-HeLa cells. Similar results were obtained with HAC#21-NIH-3T3 cells (data not shown).

We determined the transcription start site (TSS) by conducting 5′-RACE (rapid amplification of cDNA ends), using an RT primer specific to the unique sequence of the targeting vector (RT in Fig. 4B). We obtained a discrete band amplified by 5′-RACE in HAC#21-HeLa and HAC#21-NIH-3T3 cells, but not in HeLa or NIH-3T3 cells. Sequencing of the PCR product showed that the transcription started at two closely positioned nucleotides that resided within the targeting vector backbone-derived sequence in HAC#21-HeLa cells, which were 2.3-kb proximal to the telomere repeat DNA (shown as blue circles in Fig. 4B and 4C). Interestingly, inspection of the nucleotide sequence around the TSS's identified a “tataaa” sequence 27 nt upstream of the first transcription site (Fig. 4C). This TATA-box-like sequence may have specified initiation of transcription of the telomeric repeat-containing RNA, which hereafter we refer to as HAC-telRNA.

The C-terminal domain (CTD) of the largest subunit of RNA polymerase II (Pol II) complex contains tandem repeats of seven amino acids (the consensus sequence is YSPTSPS) in a wide variety of eukaryotic species. The serine and threonine residues of the heptad repeat are phosphorylated depending on the step at which the Pol II is committed in transcription [33]. In a simplified view, phosphorylated Ser-5 (S5P) of CTD is most abundant when Pol II transcribes the 5′-end of genes. Accordingly, S5P is enriched at active promoters. In contrast, phosphorylation of Ser-2 (S2P) is abundant with processively elongating Pol II. We examined the CTD phosphorylation of Pol II associated with the DNA region encoding HAC-telRNA in ChIP experiments using antibodies specific to S2P or S5P phosphorylated Pol II [27], or Pol II irrespective of its CTD phosphorylation status (Fig. 4D). In control experiments, we found that S5P-Pol II was highly enriched in the region about 0.1-kb downstream of the TSS of the γ-actin gene (*ACTG1*), as expected. We could not obtain workable real-time PCR primers for the region immediately downstream of the HAC-telRNA TSS for technical reasons. We therefore conducted Pol II ChIP experiments using a primer set amplifying a region 0.2-kb upstream of the TSS (region w in Fig. 4D). We detected binding of Pol II S5P at the region w, albeit to a lesser extent than at the TSS of *ACTG1*, suggesting that transcription of HAC-telRNA started from a region close to the region w. The absence of S5P Pol II at the regions x or y suggests that there is not an alternative TSS for HAC-telRNA near the telomere repeats on HAC#21. In contrast, Pol II S2P, a mark for elongating Pol II, was present at the regions x and w. Collectively, these results indicate that Pol II initiates transcription of HAC-telRNA at TSS's within the integrated targeting vector sequence, and elongation proceeds toward the telomere repeats.

**Figure 5:**
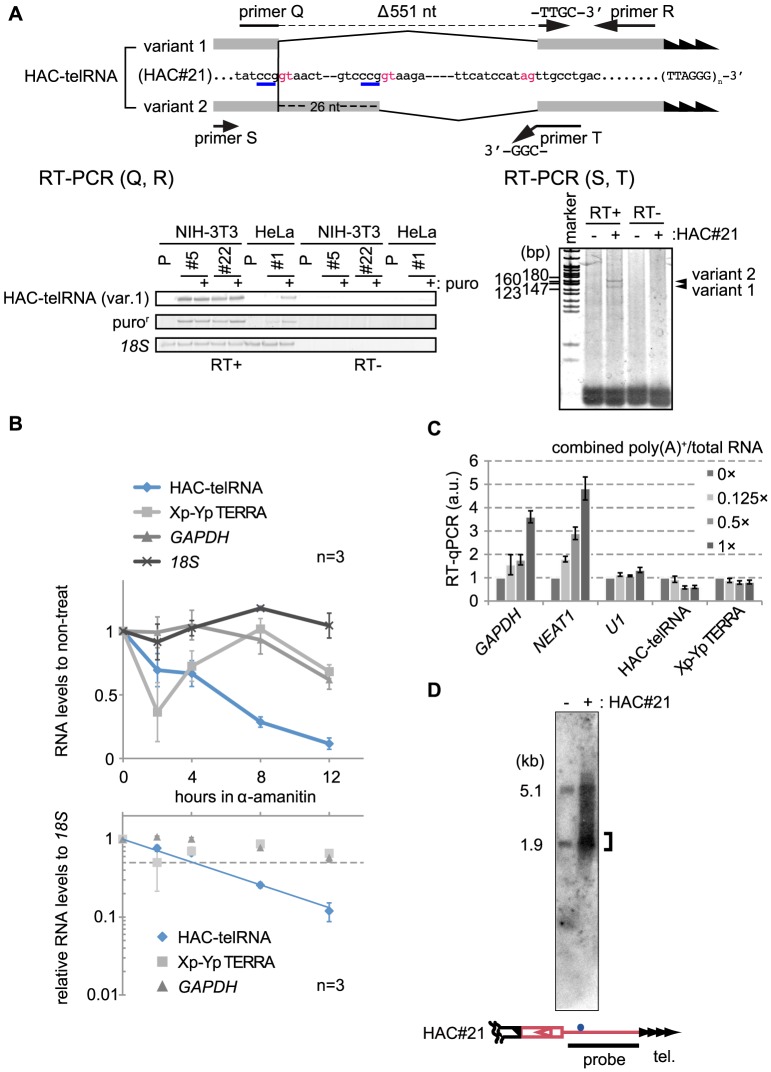
The telomeric transcript from HAC#21, HAC-telRNA, is an unusual Pol II transcript. A. Splicing of HAC-telRNA examined by RT-PCR. (Top) exon and intron sequences of two variant transcripts are shown (exons in shade) with highlighting of the intron donor and acceptor consensus sequences in red. Primer Q spans the splice junction of two exons (broken line). Primer T ends with CGG-3′, which anneals with the last three nucleotides (GCC-3′, blue underlines)) of both variant exons. (Bottom left) HAC-telRNA variant 1 was detected using variant 1-specific primers (primers Q+R). P, parental line. #5, #22, and #1 are independent HAC#21-containing clones. (Bottom right) RT-PCR products generated by primers S and T. The two variants gave rise to two PCR products with or without the terminal 26-nt sequence of the first exon. P, parental cells; and #1, a HAC#21-HeLa clone. B. Pol II-dependent transcription of HAC-telRNA. HAC#21-HeLa cells were treated with 20 µg/ml of α-amanitin for the indicated times. Transcript levels at each time point determined by real-time PCR are shown relative to the non-treated condition (top panel), or after normalization to *18S* rRNA levels (bottom panel). The half-life of HAC-telRNA was estimated from the intersection of an exponential regression curve with the half value (dotted line). Bars indicate s.e.m. of three independent quantifications. C. Polyadenylation of transcripts. An aliquot of total RNA was affinity purified with an oligo-dT column. Various amounts of the poly(A)-enriched RNAs were mixed with the total RNA at the indicated ratios, and test RNAs present in the mixture were detected by real-time RT-PCR. cDNA was generated using a mixture of gene-specific primers. D. Northern blot hybridization of HAC-telRNA. Total RNA was obtained from HeLa and HAC#21-HeLa cells, and was subjected to avidin-bead affinity purification with biotinylated oligo DNA containing (CCCTAA)_5_ repeats. Bound RNAs were detected with a targeting-vector-specific probe (black bar in bottom). Bracket, concentrated signals in the detected profile.

We found that HAC-telRNA was generated in two variant forms showing different lengths (variants 1 and 2, Fig. 5A). The variants lacked overlapping genomic sequences of 551 nt and 525 nt, respectively, in their cDNA sequences. The missing sequences started with distinct nucleotides (..ATCCGgtaac.. in variant 1, and ..ACCCGgtaag.. in variant 2; lowercase letters indicate the missing region), which were 26-nt apart, and terminated at the common nucleotide (..ccatagTTGCCTGAC..). Interestingly, these start and terminal nucleotide sequences match intronic consensus donor and acceptor sequences. Based on this sequence information, we confirmed splicing of HAC-telRNA by RT-PCR using cDNA generated with the (CCCTAA)_5_ primer. We prepared two PCR primer sets (primers Q and R; and S and T in Fig. 5A). In both sets, one primer was encoded by a presumptive exon (primers S and R), and the other corresponded to a sequence that spans a putative splice junction (primers T and Q). Primer sets (Q and R) should specifically detect splice variant 1, but not variant 2 (Fig. 5A, upper panel). Indeed, the primer sets produced specific PCR products in two independent clones of HAC#21-NIH-3T3 (#5 and #22 in Fig. 5A, left lower panel) and one HAC#21-HeLa clone (#1 in Fig. 5A, left lower panel), but not in their parental NIH-3T3 or HeLa cells (P in Fig. 5A, left lower panel). Coincidentally, the 3′-most nucleotides of the presumptive upstream exons of variants 1 and 2 were CCG-3′. Because the 3′-end of primer T is CGG-3′, primer T will anneal with both variant 1 and 2 (Fig. 5A, upper panel). Thus, the primer set (S, T) will detect both variants 1 and 2. Indeed, we observed two RT-PCR products from HAC#21-HeLa cells using that primer set, and the respective sizes differed by about 26 bp, as predicted (Fig. 5A, right panel). The relative signal intensities of the two bands were similar, suggesting that HAC-telRNA splice variants 1 and 2 were produced at similar levels.

### HAC-telRNA shares features with TERRA

We asked whether HAC-telRNA showed characteristic features in common with TERRA. To verify that HAC-telRNA is transcribed by Pol II, we treated HAC#21-HeLa cells for various times with the Pol II inhibitor α-amanitin at 20 µg/ml. Total RNA was extracted for each time interval and transcript levels were determined by RT-real-time PCR (Fig. 5B). In control experiments, *18S* rRNA, which is transcribed by RNA polymerase I, was insensitive to the treatment, whereas Pol II-derived *GAPDH* transcript levels were reduced in the treated cells, confirming α-amanitin specificity in this experimental condition. We found that levels of HAC-telRNA, as well as of TERRA derived from chromosome Xp-Yp, were dramatically decreased by a 2-hour treatment with α-amanitin, suggesting that HAC-telRNA is produced by Pol II. Unexpectedly, however, Xp-Yp-derived TERRA showed an increase in transcript level at later time points (4 or 8 hr). This may suggest that TERRA can be synthesized by polymerase(s) other than Pol II.

To test this possibility, we conducted a similar experiment with 50 µg/ml of α-amanitin for 5 hours (Fig. S3), in which the Ser-2 phosphorylated form of Pol II (S2P) is largely depleted [34]. In an immunoblotting experiment using an antibody that recognizes total Pol II (Fig. S3A upper left, 0 hr), extracts from untreated cells showed multiple retarded bands with an apparent molecular mass greater than the largest subunit of Pol II (220 kDa) [27]. Among them, the intensity of the slowest band (filled circle in Fig. S3A) was significantly decreased by the 5-hour α-amanitin treatment. When a parallel blot was probed with the anti-S2P Pol II antibody (Fig. S3A, right), a band with mobility similar to the slowest band that was detected with anti-total Pol II almost disappeared in the treated cells (5 hr). These results imply that the slowest band represents the S2P form of Pol II. α-amanitin treatment reduced the amount of S2P Pol II, suggesting that it inhibited the Pol II-dependent transcription. Taken together, we found that 50 µg/ml α-amanitin treatment eliminated Ser-2 phosphorylated Pol II.

We again analyzed transcript levels by RT-real-time PCR in extracts of cells treated with 20 or 50 µg/ml α-amanitin for 5 hrs (Fig. S3B), and confirmed that *18S* rRNA levels were unchanged, but *c-myc* levels were reduced in the α-amanitin treated cells (Fig. S3B, left panel), as expected. To normalize the data obtained under different conditions, we plotted test RNA levels relative to *18S* rRNA levels respectively (Fig. S3B, right panel. The non-treatment condition is set to one). The *c-myc* transcript was hardly detectable at either 20 or 50 µg/ml α-amanitin, consistent with a rapid turnover rate of *c-myc* Pol II transcript, with a half-life of less than one hour [35]. These results indicate that Pol II transcription was specifically and effectively inhibited by the α-amanitin treatment, in agreement with the finding that wild-type Pol II is inactivated in the presence of 3 µg/ml α-amanitin in CHO cells [36]. TERRA derived from chromosome 15q showed a several-fold increase in abundance at the 5-hour time point, whereas Xp-Yp-derived TERRA showed a moderate increase with both drug concentrations, confirming that TERRA accumulates even when Pol II elongation (as judged by Ser-2 phosphorylation of Pol II) is undetectable (Fig. S3A and S3B).

To lend support to the idea that the increased levels of TERRA that we observed in the presence of α-amanitin resulted from transcriptional activity, we treated cells with Actinomycin D (ActD) (Fig. S3C), which inactivates all three RNA polymerases. We considered the *GAPDH* transcript as a *bona fide* marker for gradual decay upon transcription inhibition (Fig. 5B), and therefore plotted Xp-Yp TERRA levels relative to *GAPDH* transcript levels (Fig. S3C). Xp-Yp TERRA showed a steady, exponential mode of decay. Given the slower loss of the *GAPDH* transcript upon transcriptional inhibition, a half-life of around 4 hrs for Xp-Yp TERRA was estimated; this is an overestimate because we assumed little change in *GAPDH* transcript levels in this time frame. These results suggest that the increased amounts of TERRA at later time points in the α-amanitin-treated cells did not result from indirect effects, such as release of TERRA from RNA transcript degradation cycles. Rather, the increase is likely attributable to Pol II-independent TERRA transcription. The notion proposed here that TERRA can be synthesized by polymerase(s) other than Pol II has been already suggested from a previous finding that the half-life of TERRA is significantly shorter when measured in ActD-treated cells than in α-amanitin-treated cells [12]. Even if TERRA is transcribed by a non-Pol II RNAP, this seems to occur transiently, as levels of Xp-Yp TERRA were eventually reduced in α-amanitin-treated cells (Fig. 5B, 12-hour). By contrast, Pol II-independent (α-amanitin-resistant) transcription of HAC-telRNA was not observed (Fig. 5B).

A short half-life (as measured with ActD) is a characteristic of TERRA [11], [15]. From the graphs showing relative RNA levels after the α-amanitin treatment, which were normalized to the drug-insensitive *18S* rRNA levels (Fig. 5B, bottom panel), the half-life in HAC#21-HeLa cells was estimated to be approximately 4 hrs for HAC-telRNA, 12 hrs for the *GAPDH* transcript, and intermediate for Xp-Yp-derived TERRA, respectively. The longer half-life estimated for Xp-Yp TERRA in the α-amanitin experiment than in the above experiment (ActD-treatment) implies Pol II-independent accumulation of a subset of Xp-Yp TERRA. This result, together with the relatively short half-life, suggests that levels of HAC-telRNA are tightly regulated, as reported for TERRA, and as shown for Xp-Yp-derived TERRA in Fig. S3C.

To analyze whether HAC-telRNA is polyadenylated or not, we prepared poly(A)-enriched RNA using oligo-dT chromatography. Target RNAs were amplified in RT-real-time PCR experiments with gene-specific RT primers for input total RNA and poly(A)-enriched RNA templates. We compared the relative yield of the products obtained from each of the two templates (Fig. S4). The amount of template poly(A)-enriched RNA was adjusted to represent an equivalent amount of the total RNA template. For example, if a test RNA had both non-polyadenylated and polyadenylated forms, it would be detected in the poly(A)-enriched fraction, to a degree reflecting the fraction of the test RNA molecules possessing a poly(A) tail. *GAPDH* transcripts were detected in the poly(A)-enriched RNA fraction whereas *U1* snRNA was not, as expected from the fact that they possess and lack a poly(A) tail, respectively. We found that HAC-telRNA was present in the poly(A)-enriched RNA fraction, and therefore conclude that at least some HAC-telRNA is polyadenylated. It has been reported that only a small fraction of Xp-Yp TERRA is polyadenylated [37]. HAC-telRNA was detected in poly(A)-enriched RNA at a level similar to that of Xp-Yp TERRA (Fig. S4). Our observation of comparable degrees of enrichment of HAC-telRNA and Xp-Yp TERRA in the poly(A) RNA pool suggests that only a small fraction of HAC-telRNA is polyadenylated.

Nevertheless, the amount of RT-PCR products was larger for the poly(A)-enriched RNA than for the total RNA, raising the possibility that the relative amounts of RT-PCR products in Fig. S4 did not tell the relative population of the polyadenylated test RNA among the total test RNA. To circumvent this problem, in Fig. 5C, we performed RT-real-time PCR using total RNA alone, or mixtures of total RNA plus either 0.125×, 0.5× or 1×equivalent poly(A)-enriched RNA (1×equivalent poly(A)-enriched RNA corresponded to the amount of poly(A)-enriched RNA obtained from the total RNA tested in this experiment). The parallel reactions were run in identical reaction volumes, and the RT-real-time PCR yields were compared between the total RNA-only and the mixed samples (Fig. 5C). We reasoned that if all of the test RNA was polyadenylated and the poly(A)-enriched RNA was 100% polyadenylated, the RT-PCR yield would be accordingly increased for the mixed RNA compared to the total RNA alone. Alternatively, if the test RNA was not polyadenylated at all, the RT-PCR yields would be the same. For both the *GAPDH*, and *NEAT1* transcripts (*NEAT1* is a non-coding RNA that undergoes polyadenylation), the mixed samples yielded significantly increased RT-PCR products compared to the total RNA alone sample, in proportion to the added poly(A)-enriched RNA, suggesting that both *GAPDH* mRNA and *NEAT1* RNA are rigorously polyadenylated, as expected. In contrast, as for *U1* snRNA and Xp-Yp-derived TERRA, addition of poly(A)-enriched RNA did not increase the RT-PCR yields in a dose-dependent manner, indicating that small fractions, if any, of *U1* snRNA and Xp-Yp TERRA are polyadenylated. We found that the amount of RT-PCR products derived from HAC-telRNA did not increase when poly(A)-enriched RNA was mixed with the total RNA sample (Fig. 5C), suggesting that the polyadenylated HAC-telRNA, if any, is not abundant. Together with the results suggesting that some HAC-telRNA is polyadenylated (Fig. S4, which used poly(A)-enriched RNA alone as a template, and thus is likely more sensitive than Fig. 5C), we conclude that HAC-telRNA is inefficiently polyadenylated, as is the case for TERRA [15].

To estimate the total length of HAC-telRNA, we performed a Northern blot (Fig. 5D). Total RNAs were prepared from HeLa cells and HAC#21-HeLa cells, and separately incubated with the CCCTAA primer conjugated with biotin. The oligonucleotide along with any associated material, including telomeric repeat-containing RNA, was purified with avidin beads, denatured and analyzed by Northern blotting. A targeting vector-specific probe, which corresponds to the entire backbone sequence of the targeting vector (Fig. 5D, bottom, shown as a black bar), produced a smeared signal ranging from 0.9 to 6.7 kb in HAC#21-HeLa cells, but not in HeLa cells (Fig. 5D). Because the length of the subtelomeric region contained in the spliced HAC-telRNA is 1.8 kb, we estimated that HAC-telRNA contains a few kb of telomere repeats with an upper length limit of 3.9 kb. This suggests that a substantial part of the HAC#21 telomere repeat DNA is transcribed in HAC#21-HeLa cells (the estimated total length of the telomere repeats in the seeded telomere was 0.5 to 5.5 kb in HAC#21-HeLa cells, as described above). This result is in agreement with the observation that at least some native human telomere repeat DNA, up to several kb, is transcribed in TERRA [13]. To extract HAC#21-specific signals from the smeared signal observed with HAC#21-HeLa cells, we first measured the hybridization signals in HeLa cells, and subtracted the density from signals in HAC#21-HeLa cells. The resulting HAC#21-specific signals are shown in Fig. S5. To deduce the median length of HAC-telRNA, we conducted densitometric analysis. We divided the signal area bounded by the graph in Fig. S5 as consecutive columns with a single pixel width, and approximated their sum as the integral of the graph. The line at the median x divides the region bounded by the graph into equal halves (red line in Fig. S5). The median length of HAC-telRNA is thus approximately 3.1 kb, including 1.3 kb (3.1 kb minus 1.8 kb RNA derived from the subtelomere) of telomere repeats, with the assumption that HAC-telRNA is spliced. Interestingly, we noticed an aggregated subpopulation around 2 kb both in the original Northern blot of HAC#21-HeLa (Fig. 5D, bracket) and the subtracted image (Fig. S5, bracket). As the graph shape was asymmetric, revealing more products with lower mobilities, it is possible that transcription into the telomere DNA tends to terminate, or that HAC-telRNA is processed. In summary, HAC-telRNA contains UUAGGG-repeats and shows similar characteristics with TERRA.

### HAC-telRNA is largely chromatin-associated and its total amount increases moderately upon *TRF1* knockdown

**Figure 6:**
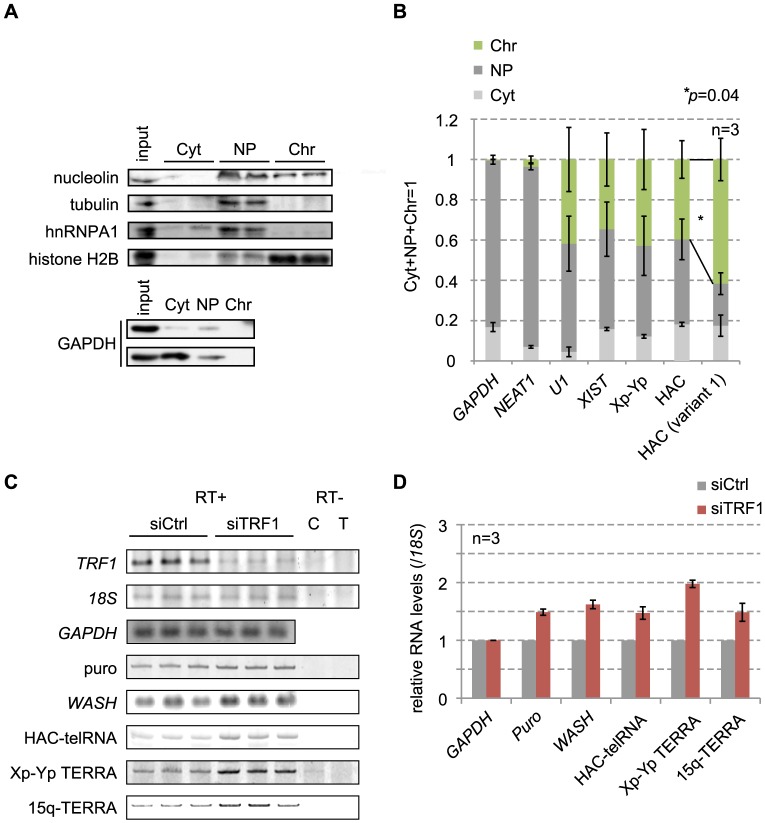
HAC-telRNA is a chromatin-associated transcript. A. Immunoblot analyses were performed for the indicated proteins to verify the efficiency of the subcellular fractionation. Cyt, cytoplasmic; NP, nucleoplasmic; Chr, chromatin. One representative result is shown. Similar results were obtained for all proteins samples used for the experiment. B. Subcellular localization of transcripts. RNAs were purified from each subcellular fraction, and the abundance of a test RNA was quantified by RT-real-time PCR. The relative abundance in each fraction is shown. Xp-Yp, Chr Xp-Yp-derived TERRA. Bars indicate s.e.m. from three independent experiments. The relative abundance of HAC-telRNA splice variant 1 (HAC (variant 1)) in the chromatin fraction was significantly larger than that of total HAC-telRNA (HAC) (asterisk, one-tailed Student's *t*-test). C. Transcript levels of indicated genes in TRF1-knockdown HAC#21-HeLa cells. Total RNAs extracted from cells at 48 hrs post-transfection were examined in triplicate by RT-PCR. A representative result is shown. D. TRF1-knockdown effect. Amounts of RT-PCR products obtained from control knockdown (siCtrl) and TRF1-knockdown (siTRF1) HAC#21-HeLa cells were quantified from SYBR Gold-stained gels. *18S* rRNA served as an internal control of three independent knockdown experiments. Bars, s.e.m. from three independent knockdown experiments.

To characterize the cellular localization of HAC-telRNA, we fractionated extracts from HAC#21-HeLa cells into cytoplasmic (Cyt), nucleoplasmic (NP), and chromatin-bound (Chr) fractions as previously reported [15]. An immunoblot showed that GAPDH, nuclear hnRNP A1 and chromatin-bound histone H2B were highly enriched in the Cyt and NP fractions, NP fraction and the Chr fraction, respectively, as expected (Fig. 6A). Tubulin was barely detected in the Cyt but was abundant in the NP fraction, probably due to polymerization of tubulin rather than incomplete extraction of the cytoplasm in our experiment. Nucleolin, which is involved in ribosome biogenesis and is typically found in both the nucleolus and the nucleus, was detected in the Chr as well as in the NP fraction. To examine whether RNAs contained in the nucleoplasm or chromatin were separated by the fractionation, test RNA present in each fraction was analyzed by RT-real-time PCR using specific primers. *GAPDH* transcripts and *NEAT1* RNA, which constitutes nuclear bodies called paraspeckles [38], were largely fractionated in the NP and Chr fractions, whereas *U1* and *XIST* RNAs were enriched in the Chr fraction, as expected (Fig. 6B). In contrast, Xp-Yp-derived TERRA was detected in the Chr and NP fractions, as reported previously [15]. We found that HAC-telRNA (detected with primers that recognized both spliced and unspliced HAC-telRNA; PCR in Fig. 4A) was also enriched in the Chr and NP fractions (Fig. 6B), suggesting that HAC-telRNA localizes to chromatin similarly to endogenous TERRA [11]. Interestingly, we observed that spliced HAC-telRNA (variant 1) was enriched in the Chr fraction to a greater extent than the total HAC-telRNA. Taken together, HAC-telRNA is largely bound to chromatin, suggesting that it functions as a non-coding RNA at the telomere.

In order to examine a possible role of telomeric proteins in the regulation of HAC-telRNA, we depleted TRF1 by siRNA and examined RNA levels two days after the transfection (Fig. 6C and 6D). The *TRF1* mRNA level was reduced to approximately 20% of the control cells, while that of *GAPDH* mRNA was unchanged, (Fig. 6C). By contrast, the abundance of HAC-telRNA, as well as endogenous TERRA derived from Xp-Yp and 15q, was moderately increased (Fig. 6D). Interestingly, expression of the puromycin resistance gene encoded by the seeded vector was also elevated. This trend was also observed for endogenous subtelomeric transcripts of *WASH*, which are transcribed from 4–6 kb proximal sites to telomere repeats in several chromosomes [39]. These results suggest that telomeric and subtelomeric transcription was derepressed when TRF1 was depleted. Collectively, we have shown that the seeded telomere in HAC#21-HeLa cells encodes a transcript that is biochemically similar to the endogenous chromosome-derived TERRA.

## Discussion

We have developed a mini-chromosome HAC#21 that is useful for analyzing the behavior of a single telomere in mammalian cells. The HAC#21 telomere was stably maintained during six weeks in HeLa cells (Fig. S1A), indicating its functional proficiency as a telomere. We have revealed that the telomere region on HAC#21 is replicated during mid-S phase in HeLa cells, that the telomere-binding proteins associate with subtelomeric regions up to 0.7-kb proximal to the telomere repeats, and that a TERRA-like non-coding RNA is transcribed from the HAC#21 subtelomere and telomere in both human and mouse cells.

### HAC#21 as a molecular tool to analyze the behavior of mammalian telomeres

HAC#21 was stable as a mini-chromosome and maintained the seeded subtelomere DNA at the chromosome end (Fig. 1). Knowing the precise nucleotide sequences of the seeded subtelomere, we were able to design primers and probes in experiments to analyze the behavior of the seeded telomere.

The seeded telomeres in HeLa and NIH-3T3 cells maintained their relative lengths, which were comparable to the endogenous telomeres of the host cells, suggesting that the seeded telomeres are under the control of the respective host cell's telomere maintenance program. A similar approach was taken to analyze telomere silencing in mouse cells [40]. Because the native telomere consists of chromatin encompassing both the telomere repeat DNA array and repetitive and polymorphic subtelomere DNAs, it is difficult to pinpoint the *cis* DNA element responsible for a telomere function of interest. The HAC technology affords an opportunity to analyze a series of telomeres composed of defined DNA sequences. In this study, we have found that an ectopic DNA region neighboring the HAC telomere repeats is transcribed. This result raises an interesting possibility that the telomere repeat DNA array *per se* may induce transcription of its upstream region, which should be tested in the future. Therefore, the current study reemphasizes the usefulness of the HAC in precisely analyzing the metabolism of a single telomere in mammalian cells.

### Replication timing of the seeded subtelomere on HAC#21 appears to be determined by the proximal native DNA of Chr21 in HeLa cells

Previous studies revealed that replication of human telomeres occur with timings specific to individual telomeres [6], [8]–[10]. Our results that the seeded telomere on HAC#21 replicates in mid-S phase in HAC#21-HeLa cells are consistent with these observations (Fig. 2).

There are few, if any, active replication origins within the telomere repeats, and telomere replication is achieved in most cases by replication forks that have fired at replication origins within the subtelomere [41]. In fiber-FISH experiments it was suggested that in human cells the replication fork, most often originating within 200 kb of chromosome ends, progresses towards telomeres at a uniform speed, and ultimately reaches telomere repeats [42].

We found that the seeded subtelomere was synchronously replicated at mid-S phase in HeLa cells (Fig. 2), similar to the targeting region in cells lacking HAC#21 (Fig. S6). Thus, the same set of origins positioned proximal to the targeted region in hChr21 fire with the same replication timing in the context of both hChr21 and HAC#21 to replicate neighboring regions. In the future, it is necessary to address the question whether the synchronous replication timing is attributed to re-setting of origin firing timings by integration of telomere repeats or just a property inherent to this specific integrated region of hChr21.

### Binding of telomere proteins decreases rapidly with distance from the telomere repeats on the HAC#21 seeded telomere in HeLa cells

In HAC#21-HeLa cells, we found that telomeric proteins were distributed to a modest extent along HAC#21 subtelomeric DNA proximal to the region 0.7-kb from the telomere repeats, but not at all in the region 3.5-kb from the telomere repeats (Fig. 3). This result in HAC#21 corroborates recent observations of native ChrXq-Yq in human HTC116 and U2OS cells [14]. Taken together, telomeric proteins TRF1 and TRF2 spread up to one kb in human subtelomeres. Because the spreading occurs to a similar degree in different sequence contexts of subtelomeres, native or seeded, it is likely based on *cis*-acting mechanisms. This contrasts with the fission yeast, in which telomere proteins can spread up to several-kb from internal sites and commit the region to heterochromatin [43], suggesting that the spreading mechanism is functionally distinct between fission yeast and mammals.

### TERRA-related RNA is transcribed from the *de novo* HAC#21 telomere in mammalian cells

In agreement with the notion that transcription through telomere DNA and/or the resulting TERRA transcript is necessary for telomeres [17], [18], we found that telomere DNA is transcribed by Pol II from a specific site downstream of a TATA-like sequence in the telomere-associated region in HAC#21, namely, the integrated vector sequence DNA. The resulting TERRA-like transcript, HAC-telRNA, shares similarity with TERRA: first, it is a chromatin-associated Pol II transcript that largely lacks a poly(A) tail and so is unstable (Fig. 5B, 5C and 6B). Second, its transcription start site resides in a similar chromosomal context, a few kb upstream of the telomere repeats (Fig. 4C). These results suggest a hypothesis that local telomere chromatin factors lead to transcription of HAC-telRNA in HAC#21. On the other hand, generation of HAC-telRNA appears to exclusively depend on Pol II (Fig. 5B), possibly reflecting promoter architecture different from that of Xp-Yp-derived TERRA.

It is believed that some TERRA have a specific CpG promoter called 61-29-37 repeats [13]. In line with a possibility that cryptic TERRA promoters specified telomere-associated transcription in HAC#21, we searched for transcription factor binding motifs shared between a region ±500 bp from the TSS for HAC-telRNA and the 61-29-37 repeats (the sequence included in a subtelomeric element called TelBam3.4 [44]). A public program for promoter searches, Proscan ver. 1.7 (http://www-bimas.cit.nih.gov/cgi-bin/molbio/proscan), identified Sp1 and CREB as candidate transcription factors, and a *cis*-element JCV-repeat was predicted in common with the 61-29-37 repeats. In contrast, the 61-29-37 repeats seemed TATA-less. Considering that half of all TERRA species including Xp-Yp TERRA are generated without the predictable 61-29-37 repeats, it is quite likely that heterologous mechanisms specify transcription of the TERRA family of RNAs, including HAC-telRNA.

There is a discrepancy concerning how the more distal telomere repeats are transcribed into TERRA. This is important because TERRA-bound proteins are involved in G-tail function related to end-protection [18]. In one report, because TERRA contains the UUAGGG tract length of 200 nt as revealed by reverse-transcription primer extension, the depth of transcription into telomere repeat DNA appears quite small compared to the several kb or more of human telomere DNA tracts [15]. In another, TERRA-specific UUAGGG tracts were up to several kb when determined by Northern blotting [13]. The larger estimate is supported by the observation that in cells over-expressing telomerase, TERRA gradually becomes elongated as the template telomere DNA is further lengthened [45]. Here, because we know the precise nucleotide length from HAC-telRNA to pure TTAGGG repeats in HAC#21, our Northern experiments demonstrate that transcription, once started, does proceed into a substantial stretch of telomere repeats (Fig. 5D).

Another novel aspect of HAC-telRNA is that it is spliced, probably at telomere chromatin. Although it is unknown whether TERRA undergoes splicing, splicing-factors were indeed detected at telomeres by mass spectrometry analyses [46], [47]. Their roles at telomeres will be addressed in the future.

Reports on regulation of TERRA by shelterin components are conflicting. It was at first addressed under the hypothesis that TERRA is a telomerase inhibitor, thereby impacting telomere length; the reportedly decreased TERRA levels upon TRF1-knockdown implied it as an effector of telomere-bound TRF1, a *cis*-acting indirect inhibitor of telomerase, in mouse cells [12]. However, no apparent effect on TERRA levels was found in cells conditionally knocked-out for TRF1 [41], suggesting that TRF1 itself is not a direct regulator of TERRA. However, we found that depletion of TRF1 via siRNA led to increased expression of different classes of RNA: telomere-related non-coding RNA (TERRA and HAC-telRNA) and subtelomeric protein-coding transcripts (*WASH* and puromycin-resistance genes) (Fig. 6C and 6D). Since the TSS's of the two types of transcripts are located similarly several-kb apart from telomere repeats, and since TERRA expression is epigenetically regulated [12], [48], a scenario can be posited in which TRF1 affects TERRA status via epigenetic repression of transcription. Our result (day 2 after siRNA treatment) can be interpreted as an early response of telomeres to TRF1-depletion before it is translated into length homeostasis (*i.e.*, to be elongated), tempting us to speculate that TERRA is a conserved regulator of telomerase [49].

Collectively, our study indicates that HAC is a useful tool to study the behavior of an individual telomere in mammalian cells. Further studies using the system will provide insights into telomere biology in humans.

## Supporting Information

Figure S1 **HAC#21 in HeLa and NIH-3T3 cells.** A. Copy number of HAC#21 retained in HeLa cells. Interphase cells were hybridized with the alphoid probe specific to hChr21 and hChr13, and the number of nuclear dot signals was counted. +puro and -puro indicate the cells cultured for 6 weeks with or without puromycin, respectively. B. The targeting vector was detected with a hChr21-specific probe (21a, 7.5 kb) and the vector-specific probe (puro, 6.3 kb). Locations of the probes used in this experiment are shown above. Southern blot hybridization using the two probes is shown below. HeLa, parental HeLa cells, and clone#1, one of the independent HAC#21-HeLa clones. Arrow indicates the 7.5-kb band specifically detected in HAC#21-HeLa cells. C. Bulk telomere DNA lengths in HeLa and NIH-3T3 cells were detected by Southern hybridization using the (CCCTAA)_4_ probe. Experiments were performed with duplicate samples (a and b). P, parental. #4 and #22, two independent HAC#21-NIH-3T3 clones.
(EPS)

Figure S2 **Specificity of anti-TRF1, TPP1, and TRF2 antibodies.** A. Whole cell extracts from HeLa or NIH-3T3 were immunoblotted with the indicated antibodies used in Fig. 3. Arrowheads indicate bands expected from the mobility of cognate proteins. B. Sonicated DNAs were de-crosslinked, purified and separated on agarose gel, followed by EtBr staining.
(EPS)

Figure S3 **Effects of RNA polymerase inhibition on TERRA transcript levels.** A. HAC#21-HeLa cells were treated with or without 50 µg/ml of α-amanitin for 5 hrs. Whole cell lysates obtained from untreated (α-amanitin 0 hr) or treated cells (5 hr) were immunoblotted with Pol II-specific antibodies. The uppermost band in the left panel (filled circle) showed lower intensity after treatment with α-amanitin, compared to the untreated cells. This band was positive with the anti-S2P Pol II antibody (right panel). Histone H2B served as a loading control. B. HAC#21-HeLa cells were treated with α-amanitin for 5 hrs at a concentration of 0, 20, or 50 µg/ml. Total RNAs were extracted from the cells and transcript levels were analyzed by RT-real-time PCR. (Left) Transcript levels are shown, with those of non-treated cells set to one. (Right) Transcript levels relative to those of *18S* rRNA are shown. The values for the lanes indicated by asterisks are as follows. *, 0.00029, 0.097, 0.025 and 0.021 (left to right); and **, 0.047, 0.015 (left to right). C. HAC#21-HeLa cells were treated with 5 µg/ml actinomycin D up to 8 hrs. Transcript levels of Xp-Yp TERRA and *GAPDH* were determined by RT-real-time PCR. Levels of Xp-Yp TERRA were normalized to those of *GAPDH* transcripts at each time point (y-axis in log scale). Half-life for Xp-Yp TERRA is estimated to be about 4 hours (dotted line), given a negligible decrease in *GAPDH* transcript levels during the experimental time-scale.
(EPS)

Figure S4 **Polyadenylation of HAC-telRNA.** Two equal aliquots of total RNA were subjected to RT-real-time PCR analysis, one of which was processed in advance for poly(A)-enrichment by using the oligo-dT column (poly(A)-enriched). The amounts of test RNAs determined by real-time interpolation were plotted as a relative ratio to those in the unprocessed aliquot of total RNA (input total RNA). Bars indicate s.d. of three independent poly(A)-purification experiments. The value of the lane indicated by an asterisk is 0.0010.
(EPS)

Figure S5 **HAC#21-specific Northern blot hybridization signals.** The signal intensity in Northern blot hybridization (Fig. 5D) was measured along each lane with a densitometer. Signal intensities in the HeLa lane were subtracted from those in the HAC#21-HeLa lane side by side, to extract HAC#21-specific hybridization signals. The resulting density profile was plotted along the distance from the top of the membrane. The median length of HAC-telRNA estimated from the signal profile is indicated by the vertical red line. The bracket indicates a noticeable aggregate of signals corresponding to a transcript length ranging from 2 to 3 kb.
(EPS)

Figure S6 **BrdU incorporation of Chr21 in S phase in HeLa cells.** A. FACS profile of DNA content in HeLa cells released from a double thymidine-aphidicolin block. B. Cumulative plots of BrdU-incorporated fractions for a test locus for the indicated hrs, determined as in Fig. 2C. The location of PCR primers around the hChr21-targeting region is shown below (Chr21).
(EPS)
