# Correction: A Human Artificial Chromosome Recapitulates the Metabolism of Native Telomeres in Mammalian Cells

**DOI:** 10.1371/journal.pone.0098825

**Published:** 2014-05-22

**Authors:** The PLOS ONE Staff

Figure 5 is incorrect in the PDF version of this article. The publisher apologizes for this error. The authors have provided a corrected version of Figure 5 here.

**Figure 5:**
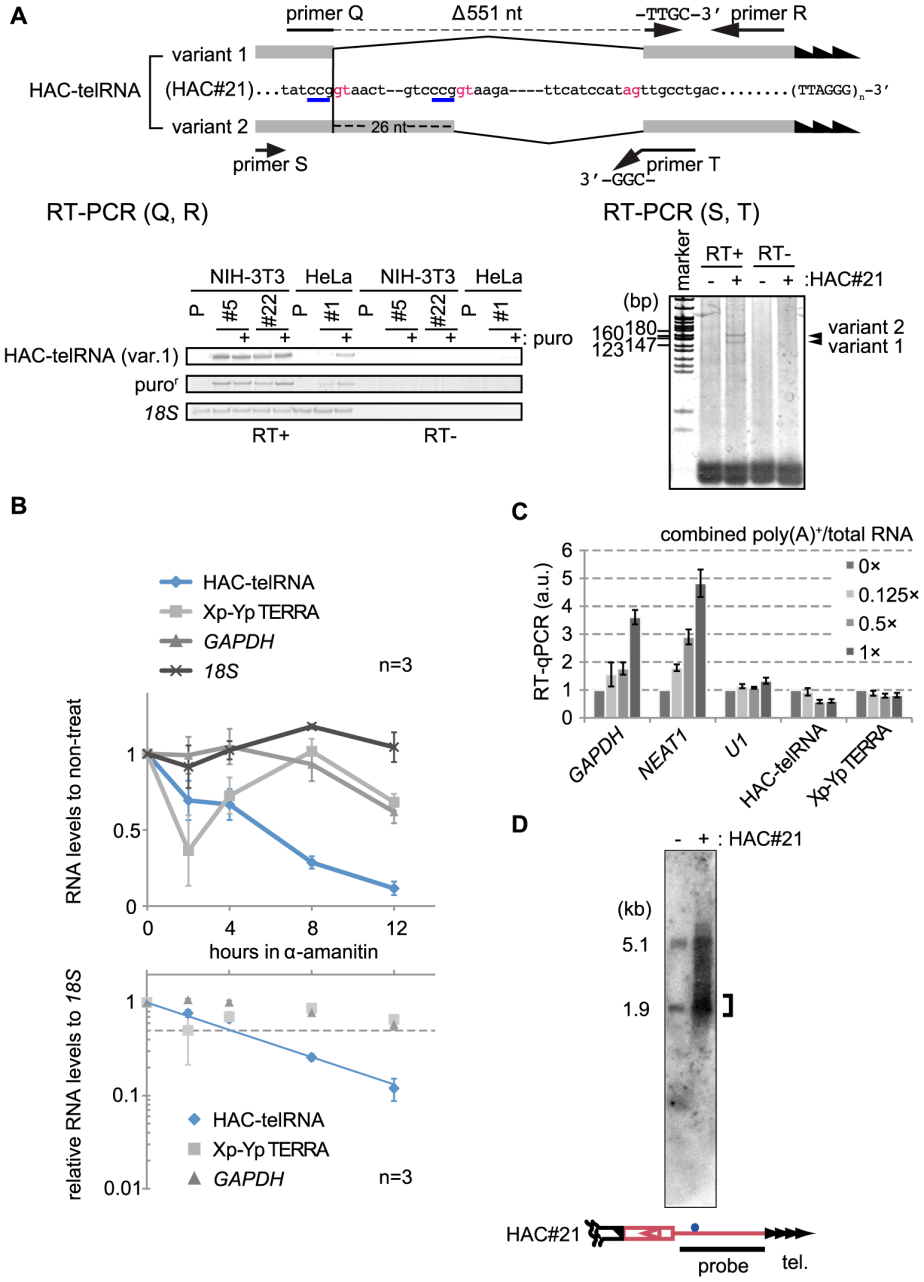
The telomeric transcript from HAC#21, HAC-telRNA, is an unusual Pol II transcript. A. Splicing of HAC-telRNA examined by RT-PCR. (Top) exon and intron sequences of two variant transcripts are shown (exons in shade) with highlighting of the intron donor and acceptor consensus sequences in red. Primer Q spans the splice junction of two exons (broken line). Primer T ends with CGG-3′, which anneals with the last three nucleotides (GCC-3′, blue underlines)) of both variant exons. (Bottom left) HAC-telRNA variant 1 was detected using variant 1-specific primers (primers Q+R). P, parental line. #5, #22, and #1 are independent HAC#21-containing clones. (Bottom right) RT-PCR products generated by primers S and T. The two variants gave rise to two PCR products with or without the terminal 26-nt sequence of the first exon. P, parental cells; and #1, a HAC#21-HeLa clone. B. Pol II-dependent transcription of HAC-telRNA. HAC#21-HeLa cells were treated with 20 µg/ml of α-amanitin for the indicated times. Transcript levels at each time point determined by real-time PCR are shown relative to the non-treated condition (top panel), or after normalization to *18S* rRNA levels (bottom panel). The half-life of HAC-telRNA was estimated from the intersection of an exponential regression curve with the half value (dotted line). Bars indicate s.e.m. of three independent quantifications. C. Polyadenylation of transcripts. An aliquot of total RNA was affinity purified with an oligo-dT column. Various amounts of the poly(A)-enriched RNAs were mixed with the total RNA at the indicated ratios, and test RNAs present in the mixture were detected by real-time RT-PCR. cDNA was generated using a mixture of gene-specific primers. D. Northern blot hybridization of HAC-telRNA. Total RNA was obtained from HeLa and HAC#21-HeLa cells, and was subjected to avidin-bead affinity purification with biotinylated oligo DNA containing (CCCTAA)_5_ repeats. Bound RNAs were detected with a targeting-vector-specific probe (black bar in bottom). Bracket, concentrated signals in the detected profile.

## References

[1] Wakai M, Abe S, Kazuki Y, Oshimura M, Ishikawa F (2014) A Human Artificial Chromosome Recapitulates the Metabolism of Native Telomeres in Mammalian Cells. PLoS ONE 9(2): e88530 doi:10.1371/journal.pone.0088530 24558398 10.1371/journal.pone.0088530 PMC3928237

